# Spatiotemporal dynamics of γH2AX in the mouse brain after acute irradiation at different postnatal days with special reference to the dentate gyrus of the hippocampus

**DOI:** 10.18632/aging.203202

**Published:** 2021-06-23

**Authors:** Feng Ru Tang, Lian Liu, Hong Wang, Kimberly Jen Ni Ho, Gautam Sethi

**Affiliations:** 1 Radiation Physiology Lab, Singapore Nuclear Research and Safety Initiative, National University of Singapore, Singapore 138602, Singapore; 2 The School of Basic Medicine, Health Science Center, Yangtze University, Jingzhou 434023, Hubei, China; 3 Department of Pharmacology, Yong Loo Lin School of Medicine, National University of Singapore, Singapore 117600, Singapore

**Keywords:** γH2AX, radiation, spatiotemporal dynamics, brain, dentate gyrus

## Abstract

Gamma H2A histone family member X (γH2AX) is a molecular marker of aging and disease. However, radiosensitivity of the different brain cells, including neurons, glial cells, cells in cerebrovascular system, epithelial cells in pia mater, ependymal cells lining the ventricles of the brain in immature animals at different postnatal days remains unknown. Whether radiation-induced γH2AX foci in immature brain persist in adult animals still needs to be investigated. Hence, using a mouse model, we showed an extensive postnatal age-dependent induction of γH2AX foci in different brain regions at 1 day after whole body gamma irradiation with 5Gy at postnatal day 3 (P3), P10 and P21. P3 mouse brain epithelial cells in pia mater, glial cells in white matter and cells in cerebrovascular system were more radiosensitive at one day after radiation exposure than those from P10 and P21 mice. Persistent DNA damage foci (PDDF) were consistently demonstrated in the brain at 120 days and 15 months after irradiation at P3, P10 and P21, and these mice had shortened lifespan compared to the age-matched control. Our results suggest that early life irradiation-induced PDDF at later stages of animal life may be related to the brain aging and shortened life expectancy of irradiated animals.

## INTRODUCTION

Gamma H2A histone family member X (γH2AX) is a phosphorylated H2AX on serine 139. While the number of its initial foci increases in lymphocytes immediately after radiation exposure, it is reduced with deoxyribonucleic acid (DNA) double-strand break (DSB) repair after acute radiation exposure. However, some fractions of foci may persist depending on radiation doses and cell types. γH2AX foci have therefore been considered as a sensitive biomarker for DSB and repair at the early stage [1, 2], and for ageing and gene silencing in unrepairable DNA damaged neurons at the late stages of radiation exposure [3–6]. γH2AX may also be involved in the brain damage and can also play a part in different neurological and neuropsychological disorders. For instance, it has been reported to be involved in the excitoneurotoxicity induced by glutamate receptor activation and seizures [7, 8], in the pathogenesis of Alzheimer's disease (AD) [9], Huntington's disease [10], and depression-related cellular senescence [11]. γH2AX has also been reported to be related to the fate of neuronal precursors at different pre- and post-natal stages of animal life including aged mice [5], as well as being involved in the regulation of necrosis in glioblastoma after irradiation [12]. A number of previous studies have suggested that radiation exposure may induce depression [13–16], AD [15, 17], and schizophrenia [15, 18]. Thus, understanding the spatiotemporal dynamics of brain γH2AX after irradiation at an early life of animal may be important for monitoring life-time radiation effect on neurodegeneration and brain aging. It can also be used for evaluating the therapeutic effects of radio-neuro-protective drugs and for designing novel therapy by targeting γH2AX protein to promote neural repair process, as well to prevent the development of neurological and neuropsychological disorders.

This study aimed to investigate the spatiotemporal dynamics of γH2AX expression in the mouse brain, in particular, dentate gyrus after acute radiation exposure. The findings may reveal 1) if there existed a significant difference in brain radiosensitivity among mice irradiated at postnatal day 3 (P3), P10 and P21; 2) where or which types of brain cells, i.e., neurons, different glial cells, epithelial cells in pia matter, cells in the blood vessel were γH2AX foci localized; 3) if irradiation induced persistent DNA damage foci (PDDF) in the brain cells at the chronic stages after radiation exposure.

## RESULTS

### Acute irradiation-induced brain γH2AX changes

### Irradiation at P3

**Figure 1:**
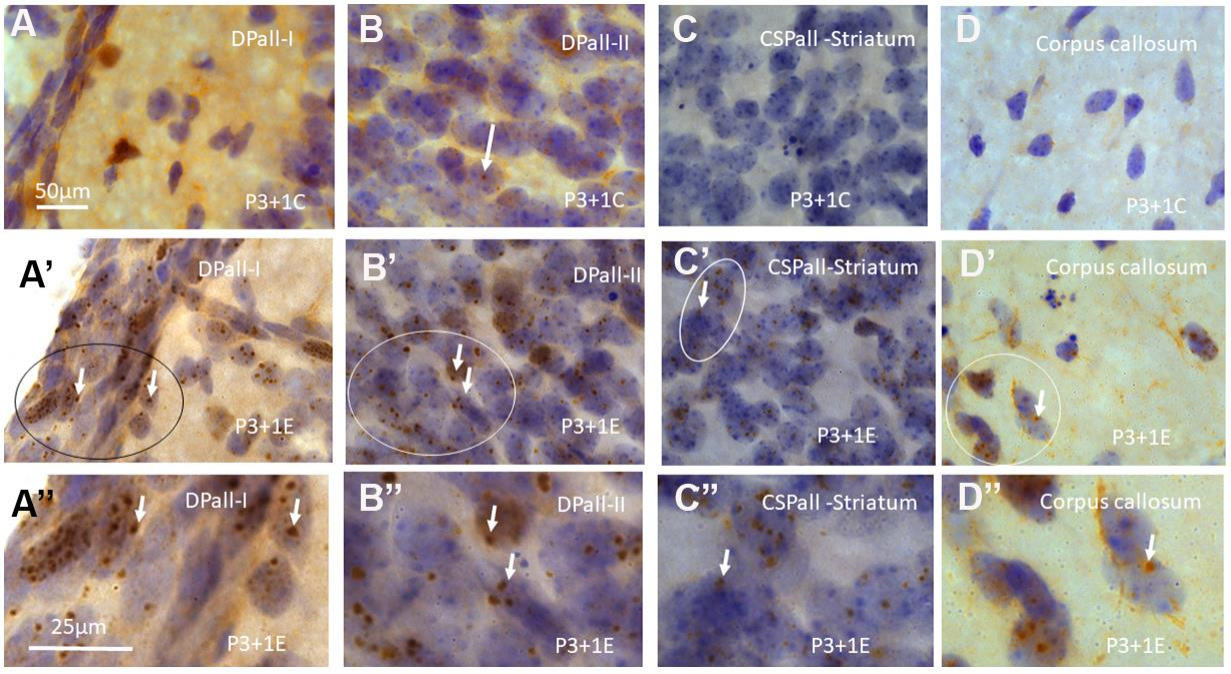
γH2AX immunostaining counterstained with hematoxylin shows very few γH2AX foci in different brain regions of postnatal day 4 (P4) mice without irradiation including dorsal pallium(DPall) /isocortex layer I (DPall-I) and pia mater (**A**), DPall –II (**B**, arrow), central subpallium/classic basal ganglia (CSPall) striatum (**C**) and corpus callosum (**D**). However, acute irradiation with 5Gy at P3 induced very significant γH2AX expression in the entire brain 1 day after radiation exposure or P4 mice. γH2AX foci could be observed in almost all brain regions at 1 day after irradiation at P3, including DPall-I (**A**’, **A**” is magnified from the ellipse in **A**’), DPall-II to DPall-VI of the grey mater (**B**’, **B**”, DPall-II, **B**” is magnified from the ellipse in **B**’), CSPall striatum (**C**’, **C**” is magnified from the ellipse in **C**’) and corpus callosum (**D**’, **D**” is magnified from the ellipse in **D**’) at 1 day after irradiation at P3. Scan bar=50μm in (**A**) applies to (**B**–**D**) (**A**’–**D**’) Scan bar=25μm in (**A**”) applies to (**B**” –**D**”).

**Figure 2:**
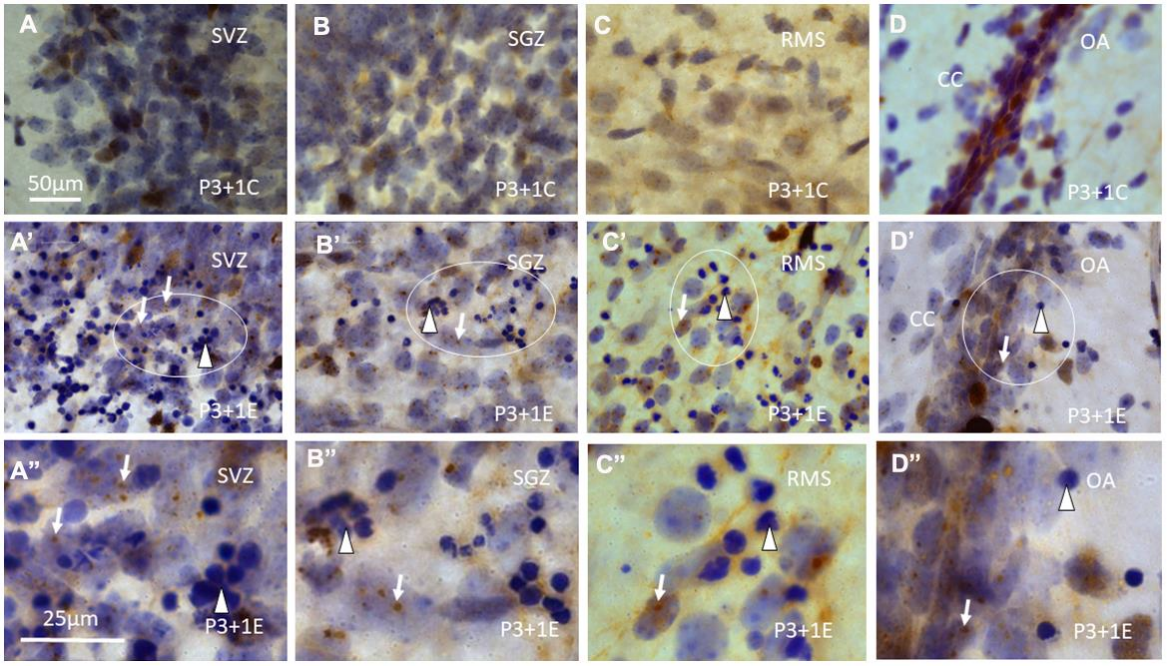
γH2AX immunostaining counterstained with hematoxylin shows that γH2AX foci are almost undetectable in the subventricular zone (SVZ) (**A**) of the lateral ventricle, subgranular zone (SGZ) (**B**), rostral migratory stream (RMS) (**C**) and the border between dorsal hippocampus (O/A: border between stratum oriens and alveus) and corpus callosum (CC) (**D**) of P4 mice without irradiation. Irradiation with 5Gy induced obvious γH2AX foci (arrows) in SVZ (**A**’, **A**” is magnified from the ellipse in **A**’), SGZ (**B**’, **B**” is magnified from the ellipse in **B**’), RMS (**C**’, **C**” is magnified from the ellipse in **C**’) and the border between O/A and CC (**D**’, **D**” is magnified from the ellipse in **D**’). Furthermore, many apoptotic bodies (arrowheads) appear in SVZ (**A**’, **A**”), SGZ (**B**’, **B**”), RMS (**C**’, **C**”) and the border between O/A and CC (**D**’, **D**”) at 1 day after irradiation at P3. Scan bar=50μm in (**A**) applies to (**B**–**D**) (**A**’–**D**’) Scan bar=25μm in (**A**”) applies to (**B**”–**D**”).

**Figure 3:**
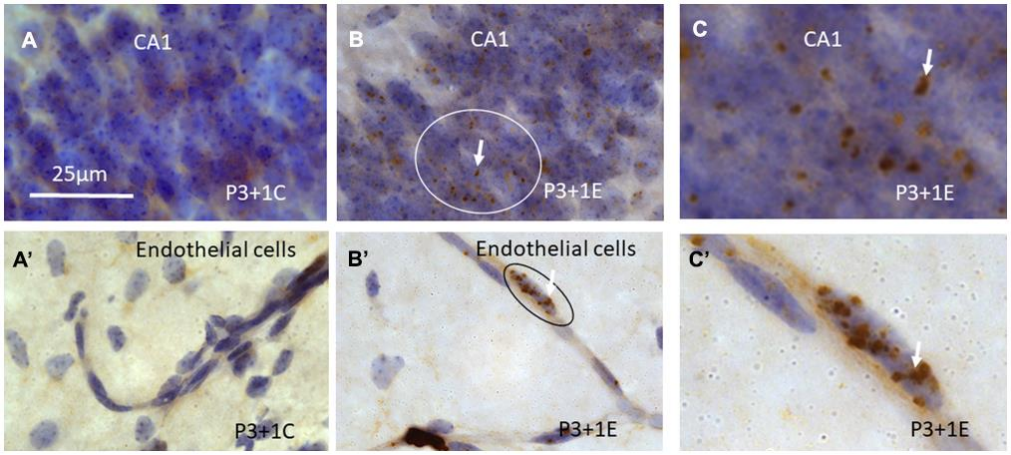
γH2AX immunostaining counterstained with hematoxylin shows that γH2AX foci are almost undetectable in the stratum pyramidale of CA1 area of the hippocampus (**A**) of P4 mice without irradiation. Irradiation with 5Gy induced obvious γH2AX foci (arrows) in the stratum pyramidale of CA1 area (**B**, **C** is magnified from the ellipse in **B**) at 1 day after irradiation at P3. Similarly, γH2AX foci are undetectable in the blood vessel of the hippocampus (A’) of P4 mice without irradiation. Irradiation induced obvious γH2AX foci (arrows) in the hippocampal blood vessel (**B**’, **C**’ is magnified from the ellipse in **B**’) at 1 day after irradiation at P3. Scan bar=25μm in (**A**) applies to (**B**–**C**) (**A**’–**C**’).

**Figure 4:**
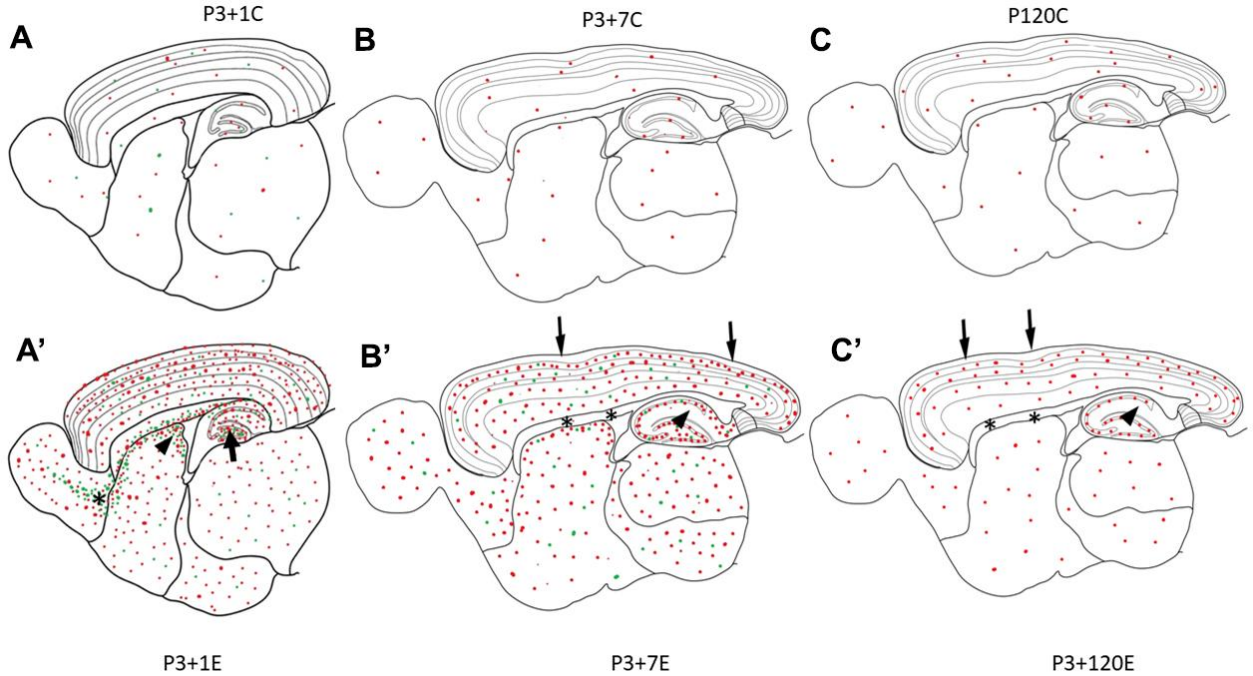
Diagrams (**A**–**C**) show γH2AX foci (red dots) in the brain of the control animal without irradiation. In P3+1C or P4 mouse brain, a few apoptotic bodies (green dots) randomly appear in different brain regions (**A**). One day after irradiation at P3, γH2AX foci (red dots) and apoptotic bodies (green dots) increase obviously in all brain regions (**A**’). Drastic increase of apoptotic bodies (green dots) appear in the hilus of the dentate gyrus (arrow), in the subventricular zone of the lateral ventricle (arrowhead) and in the rostral migratory stream (asterisk) (**A**’). Seven days after irradiation at P3, many γH2AX foci (red dots) and apoptotic bodies (green dots) still exist in all brain regions (**B**’). However, there was no obvious change of γH2AX foci in the pia mater (arrow), white matter (corpus callosum, asterisks), the strata laculosum moleculare, radiatum (arrow), oriens of CA1-3 areas, the stratum moleculare of the dentate gyrus (**B**’). One hundred-twenty days after irradiation at P3, there are still some γH2AX foci (red dots) still exist in all brain regions (**C**’) although the number of γH2AX foci is reduced obviously.

**Figure 5:**
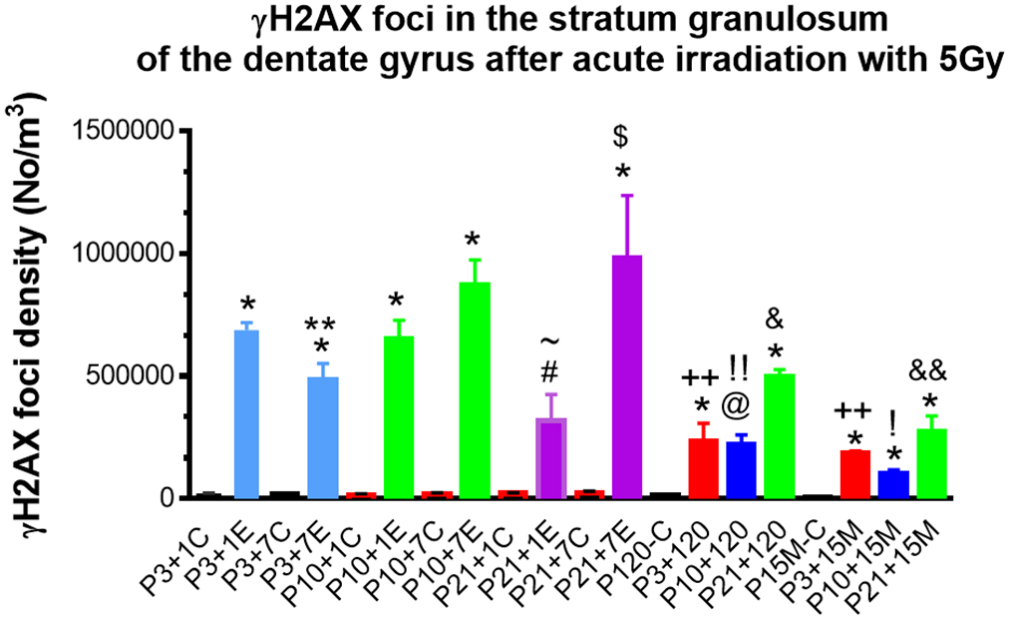
**Quantitative analysis of γH2AX foci in the stratum granulosum of the dentate gyrus among experimental mice 1day, 7, 120 days and 15 months after irradiation with 5Gy at P3, P10 and P21 respectively.** *P<0.001; @P<0.01 #P<0.05 compared to the age-matched control, &P<0.01 compared to P3+120 or P10+120; $P<0.05 compared to P21+1E; ~ P<0.05 compared to P3+1E, P10+1E; **P<0.05 compared to P3+1E and P3+7E; ++P<0.001 compared to P3+1E; !!P=0.001 compared to P10+1E; !P<0.01 compared to P10+1E; &&P< 0.05 compared to P21+120. C: Control, E: Experimental.

In the control P4 (P3+1C, i.e., 1 day after pseudo radiation exposure on P3), P10 (P3+7C, i.e., 7 days after pseudo radiation exposure on P3), P120-C (i.e., 120 days after pseudo radiation exposure on P3) mice without radiation exposure, very few γH2AX foci could be observed in different brain regions (Figures 1A–1D, 2A–2D, 3A, 3A’, Figure 4A, Figure 5) (Table 1). However, acute irradiation with 5Gy at P3 induced significant γH2AX expression in the entire brain 1 day after radiation exposure. γH2AX foci could be observed in almost all parts of brain region. It included dorsal pallium(DPall) /isocortex (from the outer pia mater, all layers of the grey matter, white matter to the subventricular zone), medial pallium (MPall) (hippocampal allocortex including the hilus, strata granulosum and moleculare of the dentate gyrus, strata laculosum moleculare, radiatum, pyramidale, oriens of CA1-3 areas), central subpallium/classic basal ganglia (CSPall), alar plate of evaginated telencephalic vesicle (TelA); prosomere 1-3 (Figure 1A’–1D’, 1A”–1D”, Figure 2A’–2D’, 2A”–2D”, Figure 3B, 3C) (refer to “The Allen Developing Mouse Brain Atlas, P4”, website: https://atlas.brain-map.org/atlas?atlas=181276162#atlas=181276162&plate=100711203&structure=15818&x=3841&y=3440&zoom=-3&resolution=7.92&z=7). γH2AX foci were demonstrated in almost all types of brain cells including epithelial cells in pia mater (Figure 1A’, 1A’’), different cells (neurons and glial cells) in the cortex (Figures 1B’, 1B”, 1C’, 1C”, 2A’–2D’’, 2A’–2D’), glial cells in the white matter (Figure 1D’, 1D”) and cells in the blood vessel (Figure 3B’, 3C’).

**Table 1:** γ-H2AX foci in the mouse brain at 1, 7, 120 days and 15 months after the acute irradiation at P3, P10 and P21.

**γ-H2AX**	**Neurogenesis and migration**				**Hippocampus**					**Cortex**			**Stri**	**Thal**	**BV**	**CC/EC**
	**SVZ**	**RMS**	**OB**	**Hilus**	**Dentate gyrus (DG)**		**CA1-3**			**I**	**II-IV**	**V-VI**				
					**SG**	**SM**	**SO**	**SP**	**SR-SLM**							
P3+1 C	+/-	+/-	+/-	+/-	+/-	+/-	+/-	+/-	+/-	+/-	+/-	+/-	+/-	+/-	+/-	+/-
P3+1 E	+++	+++	+++	+++	+++	+++	+++	+++	+++	+++	+++	+++	+++	+++	+++	+++
P3+7 C	+/-	+/-	+/-	+/-	+/-	+/-	+/-	+/-	+/-	+/-	+/-	+/-	+/-	+/-	+/-	+/-
P3+7 E	+++	+++	+++	+++	+++	+/-	+/-	+++	+/-	+++	+++	+++	+++	+++	+/-	+/-
P10+1 C	+/-	+/-	+/-	+/-	+/-	+/-	+/-	+/-	+/-	+/-	+/-	+/-	+/-	+/-	+/-	+/-
P10+1 E	+++	+	+++	+++	+++	+/-	+/-	+++	+/-	+/-	+++	+++	+++	+++	+/-	+/-
P10+7 C	+/-	+/-	+/-	+/-	+/-	+/-	+/-	+/-	+/-	+/-	+/-	+/-	+/-	+/-	+/-	+/-
P10+7 E	++	+/-	+++	+++	+++	+/-	+/-	+++	+/-	+/-	+++	+++	+++	+++	+/-	+/-
P21+1 C	+/-	+/-	+/-	+/-	+/-	+/-	+/-	+/-	+/-	+/-	+/-	+/-	+/-	+/-	+/-	+/-
P21+1 E	+	+	+++	++	+++	+/-	+/-	+++	+/-	+/-	+++	+	+++	+++	+/-	+/-
P21+7 C	+/-	+/-	+/-	+/-	+/-	+/-	+/-	+/-	+/-	+/-	+/-	+/-	+/-	+/-	+/-	+/-
P21+7 E	+	+	+++	+++	+++	+/-	+/-	+++	+/-	+/-	+++	+++	+++	+++	+/-	+/-
P+120C	+/-	+/-	+/-	+/-	+/-	+/-	+/-	+/-	+/-	+/-	+/-	+/-	+/-	+/-	+/-	+/-
P3+120	+	+	+	+/-	+++	+/-	+/-	+++	+/-	+/-	++	+	+++	+	+/-	+/-
P10+120	+	+	+	+/-	+++	+/-	+/-	+++	+/-	+/-	++	++	+++	+	+/-	+/-
P21+120	+	+	+	+/-	+++	+/-	+/-	+++	+/-	+/-	++	++	+++	+	+/-	+/-
MRI-P15M-C	+/-	+/-	+/-	+/-	+/-	+/-	+/-	+/-	+/-	+/-	+/-	+/-	+/-	+/-	+/-	+/-
MRI-P3+15M	+	+	+	+/-	++	+/-	+/-	+	+/-	+/-	+	+	+	+	+/-	+/-
MRI-10+15M	+	+	+	+/-	++	+/-	+/-	++	+/-	+/-	++	++	++	++	+/-	+/-
MRI-21+15M	+	+++	+++	+/-	+++	+/-	+/-	+++	+/-	+/-	+++	+++	+++	+++	+/-	+/-

BV: Blood Vessel; CC: corpus callosum; EC: Ependymal cells; OB: olfactory bulb; RMS: rostral migratory stream; SM: Stratum moleculare; SG: stratum granulosum; SP: stratum pyramidale; SR-SLM: Stratum radiatum and stratum laculosum moleculare; Stri: striatum; SVZ: subventricular zone; Thal: Thalamus; Per screen size under 400x for counting γ-H2AX foci: 310μm x 230μm; per screen foci: +++: > 10 foci; ++: 5-10 foci; +: 2-5 foci; +/-: 0<γ-H2AX<2. C: Control, E: Experimental.

In the subventricular zone (SVZ), rostral migratory stream (RMS) and temporal migratory stream (TMS) (Figures 2A’, 2C’, 2A”, 2C”) and olfactory bulb (OB), the subgranular zone (SGZ) of the dentate gyrus (Figure 2B’, 2B”), in the principal neurons in the strata pyramidale (Figure 3B, 3C) and granulosum and interneurons in other layers of the hippocampus, at the border between dorsal hippocampus and corpus callosum (Figure 2D’, 2D”), many γH2AX foci were also found. Hematoxylin-stained apoptotic bodies or pyknotic nuclei were observed in different brain regions, in particular, in those parts that are involved in neurogenesis and neuronal migration, i.e., SVZ, SGZ, OB, RMS and TMS (Figure 2A’–2D’, Figure 2A”–2D”).

Seven days (P3+7) after irradiation with 5 Gy at P3, many γH2AX foci still existed in the most of the brain regions. However, no γH2AX foci appeared in the pia mater, white matter, the stratum moleculare of the dentate gyrus, strata laculosum moleculare, radiatum, oriens of CA1-3 areas of the hippocampus and cerebrovascular system (Table 1).

### Irradiation at P10 or P21

One day after irradiation at P10 or P21, many γH2AX foci were observed in the layers II to VI of the DPall, in the stratum pyramidale of CA1-3 areas and stratum granulosum of the dentate gyrus in MPall (Table 1). They were also found in the brain regions involved in neurogenesis and neuronal migration, i.e., SVZ, SGZ, olfactory bulb (OB), rostral migratory stream (RMS) and temporal migratory stream (TMS) (TelA) and CSPall. Increased γH2AX foci were also demonstrated in the striatum and thalamus. However, there was no obvious change of γH2AX foci in the pia mater, white matter, the stratum moleculare of the dentate gyrus, strata laculosum moleculare, radiatum, oriens of CA1-3 areas of the hippocampus in MPall and in the cerebrovascular system (Table 1). These changes were similar to those occurred at 7 days after irradiation at P3. At 7 days after irradiation at P10, there was similar patterns of γH2AX foci distribution to 1 day after irradiation.

**Figure 6:**
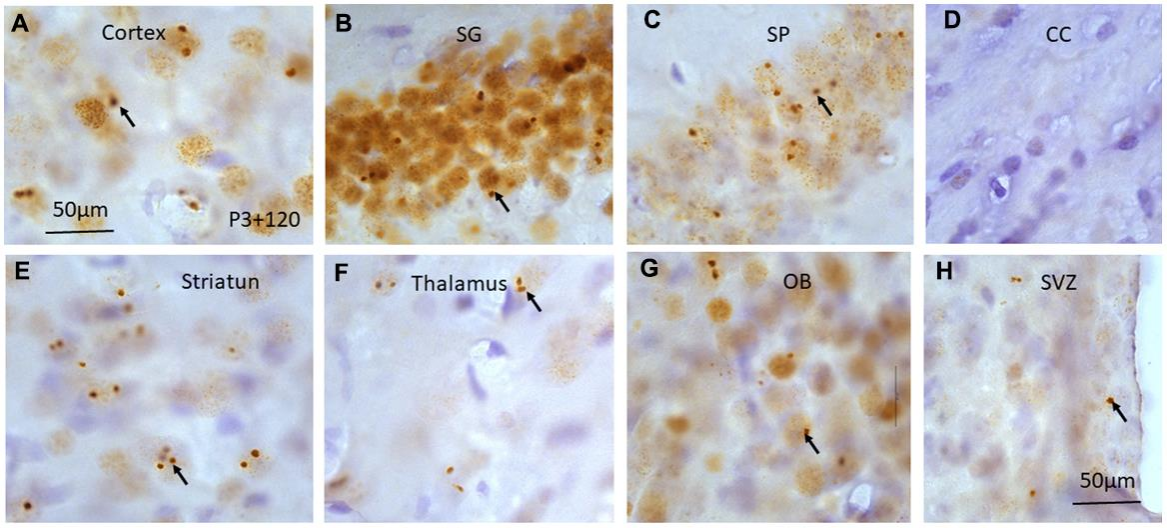
γH2AX immunostaining counterstained with hematoxylin shows irradiation-induced γH2AX foci (arrows) in different brain regions including cortex (**A**), stratum granulosum of the dentate gyrus (**B**), stratum pyramidale of CA1 area of the hippocampus (**C**), corpus callosum (CC) (**D**), striatum (**E**), thalamus (**F**), olfactory bulb (**G**) and subventricular zone of the lateral ventricle (**H**) at 120 days after irradiation at P3. Scan bar=50μm in (**A**) applies to (**B**–**H**).

Four months (120 days) after irradiation with 5 Gy at P3, P10 and P21, some γH2AX foci or PDDF could still be observed in the layers II-III of cortex (Figure 6A), the stratum granulosum of the dentate gyrus (Figure 6B), and the stratum pyramidale of CA1-3 areas of the hippocampus (Figure 6C). γH2AX foci were almost undetectable in the corpus callosum although these foci still existed in the striatum (Figure 6E), thalamus (Figure 6F), olfactory bulb (Figure 6G) and subventricular zone of the lateral ventricle (Figure 4, Figure 6H) (Table 1) although the number of γH2AX foci was decreased when compared to those at 1 and 7 days after radiation exposure.

**Figure 7:**
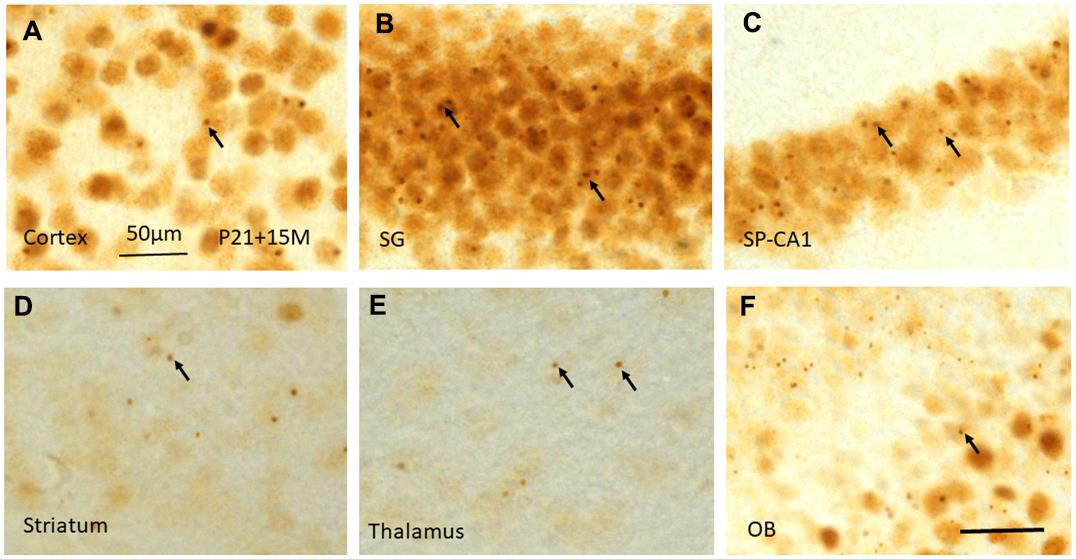
γH2AX immunostaining shows irradiation-induced γH2AX foci (arrows) in different brain regions including cortex (**A**), stratum granulosum of the dentate gyrus (**B**), stratum pyramidale of CA1 area of the hippocampus (**C**), striatum (**D**), thalamus (**E**), and olfactory bulb (**F**) at 15 months after irradiation at P21. Scan bar=50μm in (**A**) applies to (**B**–**F**).

Fifteen months (15M) after irradiation at P3, P10 and P21, similar γ-H2AX foci or PDDF could be observed as those at 120 days in the layers II-III of cortex (Figure 7A). They were also noted in the stratum granulosum of the dentate gyrus (Figure 7B), and the stratum pyramidale of CA1-3 areas of the hippocampus (Figure 7C), in the striatum (Figure 7D), thalamus (Figure 7E), olfactory bulb (Figure 7F) (Table 1). It suggested that γ-H2AX foci might be localized in those neurons involved in major brain activities such as sensation, locomotor, and learning and memory.

### γH2AX foci in the stratum granulosum of the dentate gyrus

Quantitative study of γH2AX foci in the stratum granulosum of the dentate gyrus among experimental mice 1 day, 7, 120 days and 15 months after irradiation with 5Gy at P3 indicated a significant change of the number of γH2AX foci (P<0.001 by One-way analysis of variance (ANOVA)). Student’s t-test showed a significant reduction of γH2AX foci from 1 day, 7 days to 120 days (P<0.05, by a Student’s t-test). However, no significant change in the number of γH2AX foci was observed from 120 day to 15 months after irradiation (P>0.05) (Figure 5).

The number of γH2AX foci in the stratum granulosum of the dentate gyrus among experimental mice 1 day, 7, 120 days and 15 months after irradiation with 5Gy at P10 was also changed significantly (P<0.01 by One-way ANOVA). Student’s t-test showed no significant difference in the number of γ-H2AX foci between 1 day and 7 days after irradiation at P10 (P>0.05), the number of γ-H2AX foci decreased significantly at 120 days after irradiation at P10 when compared to those at P1 and P7 day(s) after irradiation (P<0.01). From 120 days to 15 months after irradiation, the number of γ-H2AX foci in the stratum granulosum did not change significantly. There was also a significant change in the number of γ-H2AX foci in the stratum granulosum of the dentate gyrus among experimental mice 1 day, 7, 120 days and 15 months after irradiation with 5Gy at P21 (P<0.05 by One-way ANOVA). From 1 day to 7 days after irradiation at P21, the number of γ-H2AX foci increased significantly (P<0.5%). However, no difference in the number of γ-H2AX foci in the stratum granulosum at 120 days after irradiation at P21 when compared to those at P1 and P7 day(s) (P>0.05). A significant reduction of γ-H2AX foci in the stratum granulosum from 120 days to 15 months occurred (P<0.05) (Figure 5).

The number of γ-H2AX foci in the stratum granulosum of the dentate gyrus among experimental mice 1 day after irradiation with 5 Gy at P3, P10 and p21 was also changed significantly (P<0.01by One-way ANOVA). Student’s t-test showed no significant difference in the number of γ-H2AX foci at 1 day after irradiation at P3 and P10 (P>0.05). However, a significant reduction of the number of γ-H2AX foci occurred at 1 day after irradiation at P21 when compared to those at P3 and P10 respectively (P<0.05) (Figure 5).

From 120 days to 15M after irradiation at P3 and P10, no significant change of γ-H2AX foci in the stratum granulosum of the dentate gyrus was observed (P>0.05). However, a significant reduction of γ-H2AX foci from 120 days to 15M was found after irradiation at P21 (P<0.05) (Figure 5).

### Survival analysis

The long-term monitoring of 7 control, 9 P3, 7 P10, and 5 P21 irradiated mice indicated that only 1 control mouse (14%) died before the age of 13 months. However, 5 P3 (56%), 4 P10 (57%) and 2 P21 (40%) irradiated mice died before 13 months after irradiation. This finding suggested that radiation-induced brain aging indicated by γ-H2AX foci (or PDDF) may be related to shorter life expectancy in irradiated mice.

## DISCUSSION

The novel findings of this study include: 1) Acute irradiation with 5Gy induced γH2AX foci in the epithelial cells in pia mater, glial cells and blood vessel endothelial cells in the brain of mice 1 day after radiation exposure at P3, but not at P10 and P21. It suggested a drastic difference in radiosensitivity of these cells between P3 and P10 or P21 mice. Irradiation at P10 and P21 induced γH2AX foci in neurons in all brain regions at 1, 7. 120 day(s) and 15 months after irradiation with 5Gy, which was similar to those mice 7, 120 day(s) and 15 months after radiation exposure with 5Gy at P3. 2) radiation-induced γH2AX foci are mainly localized in brain neurons and persisted until the later stages (15 months after irradiation) of animal life, this PDDF may be related to the brain aging as experimental mice with PDDF died earlier than the control mice.

### Radiosensitivity of different cell types in P3, P10 or P21 mouse brain

Brain insults such as radiation exposure during critical early life stages may induce different neuropathological changes which last a lifetime, leading to the development of different brain disorders including schizophrenia [18, 19], dementia [20], depression [13], seizure [21], Alzheimer's disease [17]. The immature brain is more radiosensitive than mature one due to more neurogenesis. A number of previous studies have suggested that P10 represents the most sensitive day to different toxins including radiation exposure [22–24]. P10 mice have therefore been used to establish different brain insult models such as radiation exposure [22–24], ibotenic acid excitotoxic brain insult [25], hypoxia-ischemia [26, 27], hypoxic-ischemic encephalopathy [28]. In the present study, we showed that the epithelial cells in pia mater, endothelial cells of cerebral blood vessel and glial cells in corpus callosum in P3 mouse brain was more radiosensitive than P10 and P21 at 1 day after γ–radiation exposure with 5Gy. In the hippocampus, γH2AX foci were localized not only in principal cell layers, i.e., stratum pyramidale of CA1-3 areas and stratum granulosum, but also in the non-principal cell layers, i.e., in the strata laculosum moleculare, radiatum, and oriens of CA1-3 areas, in the stratum moleculare and hilus of the dentate gyrus 1 day after radiation exposure at P3. It suggested that γ-H2AX foci may be localized in the glial cells, cells in blood vessel and interneurons in addition to principal neurons such as pyramidal neurons in CA1-3 areas and granule cells in the dentate gyrus. The similar γ-H2AX foci in the non-principal cell layers were not observed 1 day after irradiation at P10 and P21. These observations indicate a significantly greater radiosensitivity of P3 mouse brain than P10 and P21.

At 1 day after irradiation of P3, P10 and P21 mice, there was no difference in number of γH2AX in the stratum granulosum of the dentate gyrus between P3 and P10 mice. Much fewer γH2AX foci were observed at P21 when compared to P3 or P10 mice. It suggested that P21 mouse granule cells may be less radiosensitive than P3 and P10. It is in agreement with the result indicating that granule cells in P21 mice expressed calbindin but not calretinin. The former is a mature neuronal marker whereas the latter is an immature granule cell marker [29].

A significant reduction of γH2AX foci in the stratum granulosum from 1 day, 7 to 120 days after irradiation at P3 suggested that radiation-induced DNA repair in the brain occurred continuously during these periods. However, the number of γH2AX foci did not reduce from 120 day to 15 months after radiation exposure. It suggests that with aging, radiation-induced DNA repair may become much slower, inefficient or even cease during this period. No significant reduction in the number of γH2AX foci from 1 day and 7 days, but a significant decrease at 120 days after irradiation at P10 compared to those at P1 and P7 day(s) after irradiation, it suggests that DNA repair may not be effective at early days after irradiation and granule cells may take long time to recover. On the contrary, a drastic increment in the number of γH2AX foci from 1 day to 7 days after irradiation at P21 suggested a possible delayed DNA damage and repair during this period. The different DNA repair responses indicated by γH2AX foci 1 to 7 days after radiation exposure at P3, P10 and P21 may indicate a significantly higher radiosensitivity of granule cells in earlier postnatal day mice, leading to more neuronal death and less surviving γH2AX immunopositive cells. However, the number of γH2AX foci in the stratum granulosum did not change among mice 1, 7 and 120 days after irradiation at P21 thereby suggesting that radiation-induced DNA repair in the stratum granulosum of P21 mice may occur mainly at early stage within 24 h post-irradiation. A significant reduction of γH2AX foci in the stratum granulosum from 120 days to 15 months suggested a possibly delayed DNA repair when animals were irradiated at P21. Further study may be needed to elucidate the mechanism of such a delayed DNA repair in P21 mice.

A previous study has indicated that a complete DNA repair occurred 24 h after prenatal γ- irradiation with 2 Gy at 14.5 day of gestation [30]. The presence of PDDF in the neurons of the brain in the present study suggested that further investigation is still needed to determine whether prenatal brain neurons have a high capability to self-repair or γH2AX foci still exist in the brain of animals at different postnatal stages of the animal life. A complete lost γH2AX foci was also observed in surviving neural precursor cells within 24h after irradiation [31], it is supported by our study showing the presence of γH2AX foci in the stratum granulosum, but not in the subgranular zone of dentate gyrus. However, it was inconsistent with report by Barral et al. who indicated that γH2AX in the neurogenetic regions such as subventricular zone (SVZ) of telencephalon and the cerebellar cortex was related to the fate of neuronal precursors in the adult animals [5].

### Radiation-induced γH2AX in the cerebrovascular system, hippocampal interneurons, the corpus callosum (CC) and pia mater

### Induced γH2AX in the cerebrovascular system

*In vitro* study of γ-irradiation-induced endothelial cell DNA damage, apoptosis, senescence and relevant molecular mechanisms have been well documented [32–38]. However, *in vivo* investigation of radiation-induced age-dependent DNA damages in endothelial cells of brain capillary is scarce, in particular, irradiation at different early postnatal days. *In vivo* study in the young adult rat (7-8- week-old) demonstrated abnormal changes in the blood-brain barrier (BBB) and leukocyte-endothelial cell interactions in the blood vessel in pia mater after cranial irradiation with 20Gy [39]. Acute cranial irradiation of P14 mice with 10 Gy enhanced the ratio of endothelial cells to the control in different brain regions suggesting that brain endothelial cells were more radio-resistant than other cells. It was in agreement with the finding that no significant change in Smpd1 gene from 6 h to 7 days after radiation exposure [37]. Radiation exposure with 8Gy transiently affected the hippocampal neurovascular niche in the immature brain from 6h to 1 day after irradiation [40], and there was a significant decrease in the total number of microvessels as well as the branching points from 1 to 7 weeks after radiation exposure. However, the microvessel densities were normalized with time thereafter. It suggested that from a long-term point view, radiation exposure may not affect angiogenesis of immature mouse brain [41]. Peroxisome proliferator–activated receptor gamma coactivator 1 alpha (PGC1α) is a regulator of cellular mitochondrial function and a transcriptional activator of mitochondria-related genes. It negatively regulates vascular senescence by activating reactive oxygen species detoxification and increasing the number of mitochondria [33, 42]. Radiation-induced PGC1α acetylation may be related to mitochondrial dysfunction leading to cellular senescence and aging [43]. Interestingly, irradiation of P11 rats with 6Gy induced BBB damage and reduced blood flow in the cerebellum much more than other brain regions after irradiation [44]. In the present study, γH2AX foci were observed in the endothelial cells of brain capillary 1 day, but not 7 and 120 days after irradiation with 5Gy in P3 mice, but not P10 and P21 mice. It suggested that the endothelial cells of brain capillary of P3 mice are more radiosensitive than P10 and P21 mice. The disappearance of γH2AX foci at 7 days after irradiation indicated that DNA repair process in the capillary endothelial cells might have already completed. Overall, irradiation with 5Gy may induce transient DNA damage in capillary endothelial cells in P3 mice, but may not significantly affect brain capillary in P10 and P21 mice.

### γH2AX expression in the hippocampal interneurons, the corpus callosum and pia mater

Accumulated data indicate that gamma-aminobutyric acid immunopositive interneurons are more radiosensitive than principal cells in the cortex, hippocampus, or retina after pre- or post-natal radiation exposure [45–51]. While acute radiation exposure with 6Gy to P11 rat did not affect the inhibitory network of parvalbumin interneurons in the dentate gyrus although it completely ablated neurogenesis in the subgranular zone [52]. However, acute or fractionated X-irradiation with a total of 2 to 20 Gy to mice induced a significant reduction of parvalbumin interneurons in the subgranular zone of the dentate gyrus [48–50]. Neonatal and adolescent brain insults may induce cognitive deficits and increased risk of mental illnesses, including depression, bipolar disorder, autism, epilepsy and schizophrenia. Loss of hippocampal parvalbumin interneurons may be involved in neuropathological changes of these disorders [53, 54]. In the present study, the appearance of γH2AX foci in non-principal cell layers of the hippocampus 1 day after irradiation with 5Gy in P3 mice suggested their possible cellular localization in the hippocampal interneurons and glial cells which is supported by hematoxylin counterstaining.

A number of previous studies have shown the decreased number of oligodendrocytes in the CC [55], increased density of astrocytes [56] and strongly induced inflammation-related genes such as C-C Motif Chemokine Ligand 2 (CCL2), CCL11 and IL6 [37] 120 after brain irradiation with 8 Gy to P14 mice. In the present study, the appearance of γH2AX foci in the corpus callosum 1 day after irradiation with 5Gy in P3 mice may suggest the cellular localization of γH2AX foci in oligodendrocytes. Moreover, an increased proliferation of astrocytes and neuroinflammation may support that irradiation with 5Gy may activate both astrocyte and microglia to proliferation, but not induce DNA damage or γH2AX foci in the two types of glial cells. The appearance of γH2AX foci in the pia mater at 1 day after irradiation with 5Gy at P3 suggested extensive DNA damage in epithelial cells.

### γH2AX and apoptosis

Phosphorylation of H2AX is linked not only to a DNA damage response, but also to chromatin remodelling and apoptotic DNA fragmentation [57]. Radiation-induced upregulation of pro-apoptotic genes and delayed DNA lesions in testicular cells suggests that delayed DNA lesions may be related to an active apoptotic process [58]. In neurons of prenatal radiation exposed brain, the apoptosis was related to phosphorylation and subsequent degradation of γH2AX in the course of DNA fragmentation during apoptosis [30]. The insult of a lymphocyte cell line induces γH2AX expression. However, the induction is prevented by apoptosis inhibitor, suggesting that γH2AX expression occurs subsequent to apoptosis [59]. In the present study, no co-localization of γH2AX foci with apoptotic bodies at 1 day after irradiation at different postnatal days. Combined with the previous study showing that initiation of DNA fragmentation during apoptosis results in γH2AX expression [60]. it suggests that unrepaired DNA damage or γH2AX foci at the early stages (maybe first a few hours) after irradiation with 5Gy may be related to the formation of apoptotic bodies in the mouse brain.

### Transient DNA damage foci or PDDF, aging, neurological, neuropsychological and genetic disorders

Transient expression of γH2AX has been considered as an early biomarker of neuronal endangerment after different brain insults such as glutamate receptor-dependent excitotoxicity [7, 61], seizures [8], cellular senescence [11, 62], ischemia [63], ethanol [64, 65] and radiation exposures [12, 66, 67] and genotoxic stress [68]. Some of γH2AX foci may exist in neurons as PDDF for a long period of time as shown at 15 months after irradiation in the present study. PDDF and DNA damage response (DDR) signalling are responsible for inflammatory cytokine secretion. Upregulation of PDDF and secreted IL6 occur in p53 deficient cells [69], which is causally involved in cellular senescence and organismal aging [70]. The presence of PDDF in neurons in difference brain regions and shortened life expectancy after radiation exposure at P3, P10 and P21 suggested that radiation-induced brain aging may be related to the earlier death of irradiated mice. Radiation-induced PDDF also occurred in other cell models. In MRC-5 cells, X-irradiation-induced γH2AX foci remained for 2 weeks suggesting an unrejoined DNA double-strand breaks [71]. The accumulation of PDDF of checkpoint factors may induce G1 arrest leading to tumor suppression to permanently exclude cells with remaining DNA damage [72]. We therefore speculate that the localization of PDDF in neurons may explain why radiation-induced brain tumor is mainly glia but not neuronal originated. In normal human diploid fibroblasts, X-irradiation induced transient γH2AX foci formation which disappeared rapidly. The remaining foci clustered along the chromosomal bridges 96 h after irradiation, and might be an aberrant chromatin structure by illegitimate rejoining. Large γH2AX foci continuously amplify DNA damage signal leading to an irreversible growth arrest [73–75]. In non-proliferating human mammary epithelial cells, iron-ion irradiation-induced PDDF may be related to limited availability of double-strand break (DSB) repair pathways in G0/G1-phase [76]. The increase in the size of individual foci may be due to the spreading of γH2AX over a large chromatin domain leading to the accumulation of a multitude of DNA damage response proteins distal to the lesion [77].

The defective DNA repair in neurons with accumulation of PDDF and loss of genome integrity may contribute to aging and many neurodegenerative disorders such as amyotrophic lateral sclerosis (ALS), Parkinson's disease (PD), AD and Huntington's disease (HD). In lumbar motor neurons from ALS patients, a significant up-regulation of γH2AX, phosphorylated ataxia telangiectasia mutated (p-ATM), cleaved poly (ADP-Ribose) polymerase 1 (PARP-1) and tumour suppressor p53-binding protein (53BP1) was observed [78]. Intracellular accumulation of α-synuclein (α-syn) is a hallmark of synucleinopathies, including PD. Exogenous addition of preformed α-syn fibrils (PFFs) into primary hippocampal neurons induced α-syn aggregation and accumulation and increased H2AX Ser139 phosphorylation, suggesting that γH2AX may play a role in α-syn induced pathogenesis of PD [79]. Failure to engage the repair system and initiate repair after DNA damage is likely a key mechanism for determining early damage in the hippocampus of AD [80]. L5, a human plasma low-density lipoprotein (LDL), fraction induces cell damage by activating ATM/H2AX-associated DNA breakage pathway and apoptosis via lectin-like oxidized LDL receptor-1 (LOX-1) signalling to p53, leading to cleavage of caspase-3, and to the development of neurodegenerative diseases including AD [81]. However, no correlation between persistent γH2AX foci and apoptosis after irradiation with high-dose synchrotron-generated microbeams in cultured normal human fibroblasts and p53 wild-type malignant glioma cells may suggest the importance of non-apoptotic responses such as p53-mediated growth arrest or premature senescence [82].

Increased levels of γH2AX occurred not only in astrocyte [9] and neuronal [83] nuclei, but also in blood lymphocytes of AD patients, suggesting that lymphocyte γH2AX levels may be used to identify population with a high risk of AD development [84]. Increased proportions of γH2AX-labeled neurons and astrocytes in the hippocampus and frontal cortex of mild cognitive impairment (MCI) and AD patients also suggested that there might exist a causal relationship between the early neuronal accumulation of DSB and neurodegenerative disorders including HD [10] and cognitive impairment [85]. Increased γH2AX, Checkpoint *kinase* 2 (*Chk2*) protein phosphorylation, p53 were also reported in Down syndrome (DS) fibroblasts from both fetal and adult donors during unperturbed growth conditions [86], in the hippocampal neurons up to 14 days after laparotomy in elderly mice [87]. γH2AX may also be used as a reliable marker of gene silencing in DNA damaged neuron as unrepaired DNA in neurons is sequestrated in discrete PDDF of transcriptionally silent chromatin which is essential to preserve genome stability and prevent the synthesis of aberrant mRNA and protein products encoded by damaged genes [88, 89].

At molecular level, inhibition of glycogen synthase kinase 3β (GSK3β) accelerated DSB repair efficiency in irradiated mouse hippocampal neurons, which coincided with attenuation of irradiation-induced γH2AX foci. It suggested that GSK3β inhibitors might be promising radio-neuro-protectants [90]. Radiation-induced expression of pro-survival and DNA repair proteins such as γH2AX and 53BP1, and DNA repair capacities are negatively regulated by Sirtuin 2 (SIRT2) leading to cell death and DNA damage [91].

### Concluding remarks

Extensive γH2AX foci were induced in the mouse brain at 1 day after irradiation at P3, P10 and P21, which lasted till 15 months after irradiation. The appearance of γH2AX foci in the epithelial cells in pia mater, glial cells and blood vessel endothelial cells in the brain of mice 1 day after irradiation at P3, but not P10 and P21 suggested that P3 mouse brain is much more radiosensitive than P10 and P21. The early life radiation-induced γH2AX foci, or PDDF may be involved in the brain aging leading to the shortened life expectancy in irradiated animals. From a speculative point of view, PDDF may be involved in the radiation-induced progressive neuronal loss, and the onset of different neurological and neuropsychological disorders depending on the patterns of neuropathological changes. It may also represent gene silencing in DNA damaged neuron to preserve genome stability and prevent the synthesis of aberrant mRNA and protein products as suggested previously [70, 88, 89].

## MATERIALS AND METHODS

### Animal irradiation

A total of 80 mice (5 in each control and experimental groups) was used for short- to mid-term study at 1 (5xP3+1 control; 5xP3+1 experimental; 5xP10+1 control; 5xP10+1 experimental; 5xP21+1 control; 5xP21+1 experimental), 7(5xP3+7 control; 5xP3+7 experimental; 5xP10+7 control; 5xP10+7 experimental; 5xP21+7 control; 5xP21+7 experimental) and 120 (5xP120-control; 5xP3+120 experimental; 5xP10+120 experimental; 5xP21+120 experimental) days after radiation exposure. To monitor long-term (15 months) effect of acute radiation exposure and brain γH2AX and ageing, 28 mice, i.e., 7 control (including 3 P3, 2 P10 and 2 P21), 21 experimental (including 9 P3, 7 P10, and 5 P21) mice were used. The animals were irradiated according to the same protocol in our previous studies [92]. In brief, freely moving mice at postnatal day 3 (P3), P10 and P21 were whole body γ-irradiated with 5 Gy (3.33Gy/m). We chose 5Gy because with this dose, impairment of neurogenesis could be consistently induced but there is no mortality in Balb/c mice [48, 49, 51, 92]. Animals were euthanized with a mixture of ketamine (75mg/kg) and medetomidine (1mg/kg) at 0.1ml/10g 1, 7, 120 day(s), and 15 months after irradiation, and perfused with 4% paraformaldehyde. The mouse brain was removed, post-fixed overnight, and then transferred to 30% sucrose in 0.1M phosphate buffer (PB) (pH: 7.4). All the animal experimental protocols were approved by the Institutional Animal Care and Use Committee (IACUC) of National University of Singapore (R15-1576). In addition, consistent efforts were made to minimize animal suffering and to use the minimal number of animals throughout the study.

### Immunohistochemical staining

Immunohistochemical staining was done according to our previous studies [92]. Sagittal brain sections at 40μm were blocked with 3% H2O2 and then 4% normal goat serum at room temperature. The sections were incubated with primary rabbit antibody for γH2AX (1: 200) (Cell Signaling Technology, Inc, MA, Danvers, USA) and then placed in goat anti-rabbit secondary antibody for 1 hour. The sections were placed in avidin–biotin complex (ABC) reagent (Vector Laboratories Inc., Burlingame, CA, USA) for 1 h, then reacted in 3,3'-diaminobenzidine (DAB) Peroxidase Substrate (Vector Laboratories Inc., Burlingame, CA, USA) for 10 minutes. The sections were mounted, covered and the images were then taken under microscopy (Leica Microsystems GmbH, Wetzlar, Germany).

### Hematoxylin counterstaining

To further analyse cellular localization of γH2AX foci at 1, 7 and 120 day(s) after irradiation at P3, P10 and P21 respectively, coverslip was removed after stereological analysis by immersing slides in sequence in Histo-Clear, 100%, 95%, 75%, 50%, 25% ethanol, and then in ultrapure water. The sections were then counterstained with hematoxylin and covered with coverslip.

### Statistical analysis

For unbiased counting of γH2AX foci in the stratum granulosum of the dentate gyrus, the Stereologer from Stereology Resource Center Biosciences, Inc. (SRC Biosciences, Florida, USA) was used. Three (P3+1, P3+7, P10+1, P10+7) to six (P21+1, P21+7, P120-control P3+120, P10+120, P21+120, P15M-control, P3+15M, P10+15M, P21+15M) brain sections from individual mouse were counted. γH2AX foci were counted at 400×, and indicated as the number of foci/mm^3^ in the stratum granulosum of the dentate gyrus. All the data were then analyzed by one-way ANOVA followed by student’s t-test. Statistical significance was considered at P<0.05.
